# A hybrid mock circulatory loop integrated with a LED-PIV system for the investigation of AAA compliant phantoms

**DOI:** 10.3389/fbioe.2024.1452278

**Published:** 2024-10-10

**Authors:** Francesco Bardi, Emanuele Gasparotti, Emanuele Vignali, Maria Nicole Antonuccio, Eleonora Storto, Stéphane Avril, Simona Celi

**Affiliations:** ^1^ BioCardioLab, Bioengineering Unit, Ospedale del Cuore, Massa, Italy; ^2^ Mines Saint-Étienne, Université Jean Monnet, INSERM, Saint Étienne, France; ^3^ Predisurge, Grande Usine Creative 2, Saint Étienne, France

**Keywords:** LED-PIV, hemodynamics, mock circulatory loop, abdominal aortic aneurysm, aortic phantom, 3D-printing

## Abstract

**Background:**

Cardiovascular diseases remain a leading cause of morbidity and mortality worldwide and require extensive investigation through *in-vitro* studies. Mock Circulatory Loops (MCLs) are advanced *in-vitro* platforms that accurately replicate physiological and pathological hemodynamic conditions, while also allowing for precise and patient-specific data collection. Particle Image Velocimetry (PIV) is the standard flow visualization technique for *in-vitro* studies, but it is costly and requires strict safety measures. High-power Light Emitting Diode illuminated PIV (LED-PIV) offers a safer and cheaper alternative.

**Methods:**

In this study, we aim to demonstrate the feasibility of a Hybrid-MCL integrated with a LED-PIV system for the investigation of Abdominal Aortic Aneurysm (AAA) compliant phantoms. We considered two distinct AAA models, namely, an idealized model and a patient-specific one under different physiological flow and pressure conditions.

**Results:**

The efficacy of the proposed setup for the investigation of AAA hemodynamics was confirmed by observing velocity and vorticity fields across multiple flow rate scenarios and regions of interest.

**Conclusion:**

The findings of this study underscore the potential impact of Hybrid-MCL integrated with a LED-PIV system on enhancing the affordability, accessibility, and safety of *in-vitro* CVD investigations.

## 1 Introduction

*In vitro* studies play a crucial role in cardiovascular disease (CVD) research, as they represent a great alternative to animal models (Celi et al., 2022) and can serve as a reliable means to validate *in silico* methods.

Incorporating 3D models (Garcia et al., 2018) into a mock-circulatory loop (MCL) system offers the *in-vitro* test-bench solution to reproduce physiological and pathological conditions with patient-specific accuracy in terms of both geometry and hemodynamics in a fully controlled environment. Indeed, MCLs allow for the collection of precise and patient-specific data, thus providing valuable insights into biological flows that can significantly enhance our knowledge of CVDs. MCLs have been extensively used not only to test cardiovascular devices such as heart valves (Hasler and Obrist, 2018) and LVADs (Ochsner et al., 2013; Zimpfer et al., 2016; Chassagne et al., 2021), but also to study the complex fluid dynamics inside blood vessels (Kefayati and Poepping, 2013; Bonfanti et al., 2020; Mariotti et al., 2023).

In this context, Particle Image Velocimetry (PIV) (Raffel et al., 2018) is the reference flow visualization method that provides high spatial and temporal resolutions and has been used to validate *in-vivo* measurement techniques (Roloff et al., 2019). However, a standard PIV setup entails substantial costs, both in terms of initial purchase and ongoing operation and maintenance. The main cost is due to the illumination source, which is a high-power double-pulse laser, either Nd:YAG or Nd:YAF (Raffel et al., 2018). In addition, special training is required to safely operate these laser systems (classified as Class 4 lasers) as they can cause permanent blindness or skin burns.

To address these challenges, alternative illumination sources have been explored (Cierpka et al., 2021; Caridi et al., 2022), with light-emitting diodes (LEDs) emerging as a promising solution. LEDs offer a safer, more cost-effective alternative to traditional lasers, with the added benefit of easier handling and reduced maintenance costs. The feasibility of using LEDs for PIV was first demonstrated by Willert et al. (2010), who developed an electronic circuitry capable of operating LEDs with high-pulsed currents. Their work showed that by using pulse widths as short as 20 μ s, flow speeds exceeding 0.5 m/s could be accurately measured within a 50 × 50 mm^2^ field of view. Subsequent studies, such as Buchmann et al. (2012), further validated the reliability and precision of LED-PIV, particularly for tomographic measurements. In the cardiovascular domain, Geoghegan et al. (2013) applied LED-PIV to study flow within a stenosed carotid artery phantom, demonstrating its potential for hemodynamic studies. The Abdominal Aortic Aneurysm (AAA) is a pathological condition characterized by the abnormal dilation of the arterial wall. AAAs are associated with complex flow phenomena, including flow separation, vortex formation, and transition to turbulence (Egelho et al., 1999; Salsac et al., 2006b; Yip and Yu, 2001; Stamatopoulos et al., 2011; Deplano et al., 2014; Moravia et al., 2022). Understanding these flow patterns is essential for assessing the risk of aneurysm rupture and for developing effective treatment strategies.

In this work, we seek, for the first time, to extend the application of LED-PIV to the study AAAs fluid dynamics. Our primary objective is to demonstrate the feasibility of using LED-PIV for investigating the hemodynamics of large vessels. We specifically focused on the abdominal aorta as a test case due to the intricate flow dynamics induced by the presence of an aneurysm. To achieve this, we employed a Hybrid Mock Circulatory Loop (HMCL) combined with LED-PIV. HMCLs (Ochsner et al., 2013; Cappon et al., 2021; Bardi et al., 2023) distinguish themselves from traditional MCLs by incorporating an active numerical-hydraulic module, which allows for continuous and interactive tuning of the pressure boundary conditions. This capability is particularly useful when working with deformable phantoms derived from patient-specific geometries. By integrating the advanced control capabilities of the HMCL with the LED-PIV setup, we present a novel approach for investigating AAA hemodynamics. Through validating the effectiveness of LED-PIV in this context, we aim to highlight its broader potential in cardiovascular research, particularly in enabling safer and more cost-effective experimental studies.

## 2 Methods

### 2.1 Fabrication of AAA models

Two deformable transparent models of AAA morphology were manufactured using the “lost-mold casting”: an idealized axial-symmetric AAA model and a patient-specific AAA model. This process consisted in 3D-printing internal and external molds in a water-soluble material, which were dissolved in hot water once the casting material had cured. Each step involved in the lost-mold casting is described in detail in Antonuccio et al. (2023) and is summarized herein.

**FIGURE 1:**
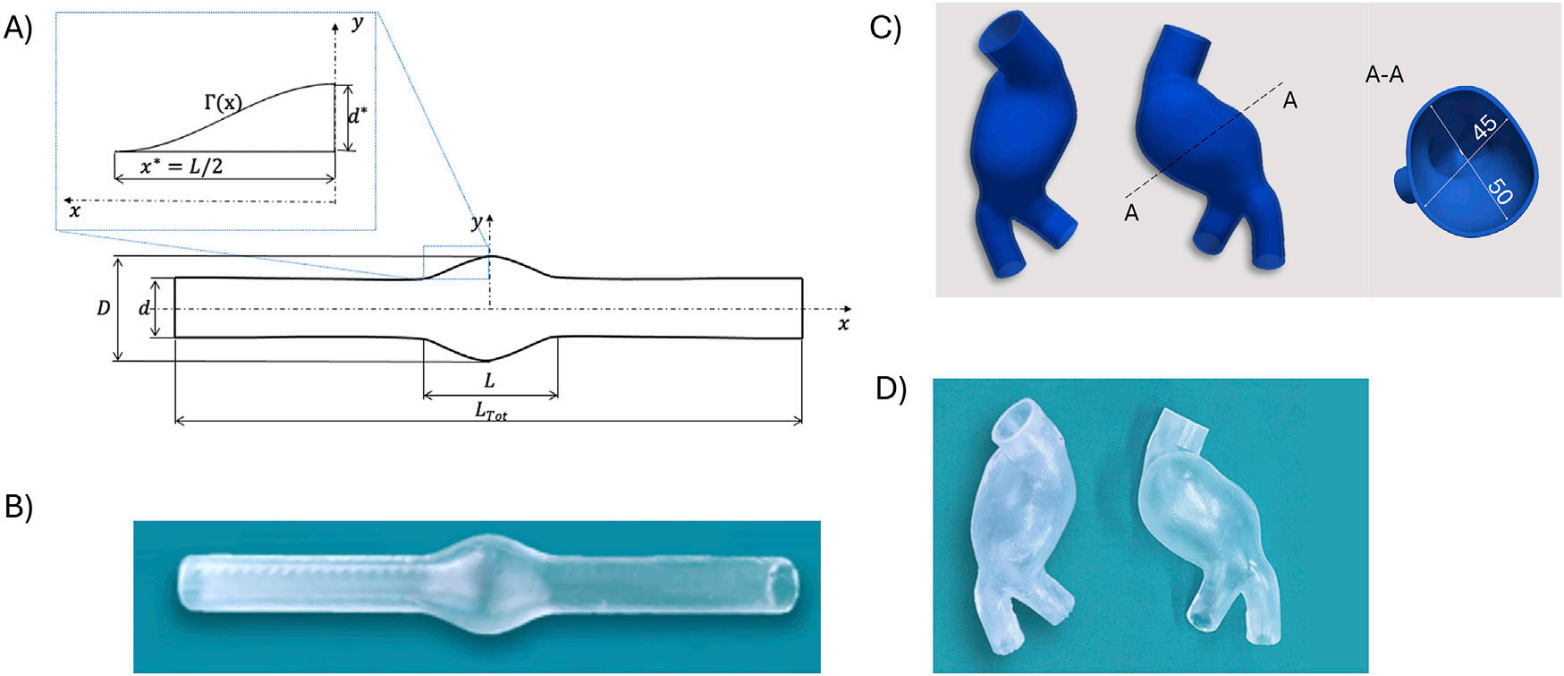
Representations of the idealized axial-symmetric model **(A)** and the associated deformable phantom **(B)**. CAD representations of the patient-specific AAA model **(C)** and associated patient-specific phantom **(D)**.

The manufacturing process comprised six key steps: *i)* model creation; *ii)* mold design; *iii)* 3D-printing of the molds; *iv)* surface treatment; *v)* material casting; *vi)* removal of the molds. The idealized axial-symmetric AAA model was developed based on a geometry employed in Salsac et al. (2006a) for fabricating rigid models. Two geometrical parameters define the AAA shape: 1) the aspect ratio L/d (where L is the length of the aneurysm bulge, and d is the inner diameter of the healthy parent vessel); and 2) the dilatation ratio D/d (where D is the maximum diameter of the aneurysm bulge). The model’s geometrical characteristics are as follows: d = 20 mm, $L_{\mathrm{Tot}}$ = 250 mm, L/d = 2.9, and D/d = 1.9. The parametric curve Γ(x) (shown in Figure 1A) that define the shape of the bulge is the following:

$$\left\{\begin{array}{cc} x^{*}=0.5\left(L/d\right)/d\;\\ d^{*}=0.5\left(D/d\right)-\left.1\right)d\;\\ \Gamma \left(x\right)=d^{*}\cos\left(\pi x/2x^{*}\right)+d/2,\; & x\in \left[-x^{*},x^{*}\right] \end{array}\right.$$

Figure 1A depicts the CAD representation of the idealized axial-symmetric AAA model.

Regarding the patient-specific model, computed tomography scans of a patient with AAA were segmented to reconstruct the 3D vessel geometry, including the abdominal aorta, aneurysm bulge, and left/right iliac (Antonuccio et al., 2023). The segmented geometry was exported as a stereo-lithography format file, converted into a solid model, and further processed in SOLIDWORKS (Dassault Systèmes S. A., Vélizy-Villacoublay, France) to design molds using the same approach for both AAA models.

A wall thickness of 1.8 mm was obtained by applying an outward offset to the inner surface of the models. A core and two outer molds were designed for material casting. The core, defining the inner surface, featured a hollow design with 15 mm reference pins at inlets and outlets.

Outer molds, defining the outer surface, were created through a multi-step process involving the creation of a monolithic external mold, splitting it into two counterparts, making the outer molds hollow by subtracting the core and solid model, and designing sprues, reference pins, and gluing channels.

All molds were 3D-printed using a fused deposition modeling printer (A4v3, 3NTR Italy) with a 2.85 mm polyvinyl alcohol (PVA) filament. PVA, a water-soluble material, dissolved upon curing of the casting material. Before casting, mold surfaces in contact with the casting material underwent treatment to eliminate roughness. Molds were assembled, closed firmly, and cast with two-component silicone Sylgard 184 (Dow, Wiesbaden, Germany), known for its compliant properties, which are suitable for vascular district fabrication for optical applications Yazdi et al. (2018). The material’s compliance aligns with the elasticity range of the human aorta, which varies between 0.25 MPa and 1.7 MPa, as reported by Lang et al. (1994). After silicone curing for 48 h at room temperature, the PVA components dissolved by immersing the molds in warm water in an ultrasonic tank. Four washing cycles, including a 1-h ultrasound-enabled cycle at 60°C, were performed. The dissolved PVA left a smooth lumen and a smooth exterior surface. The resulting deformable transparent models were subsequently soaked in cold water for finalization (Figures 1C, D).

### 2.2 Mock circulatory loop

**FIGURE 2:**
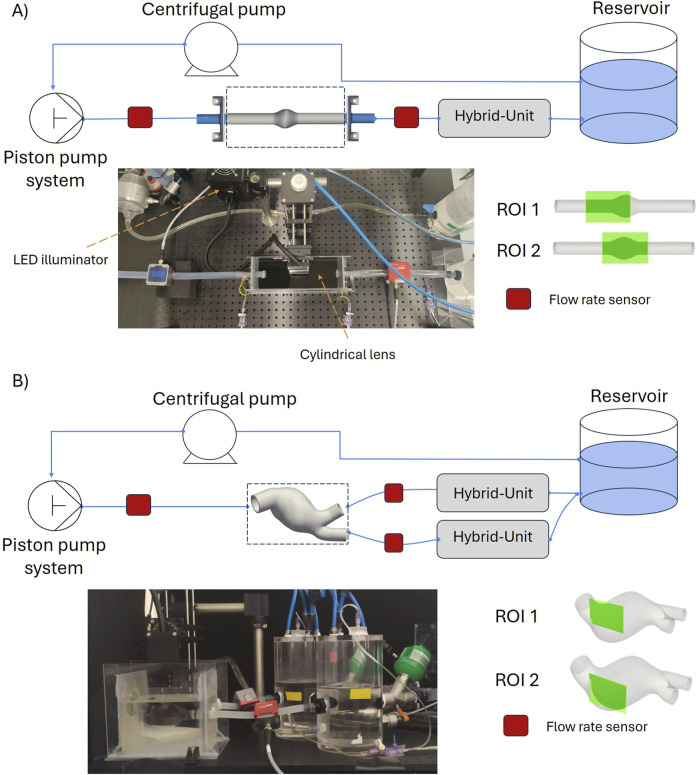
Hybrid Mock Circulatory Loop set up for the idealized AAA model **(A)** and for the patient-specific AAA model **(B)**.

A flow circuit was assembled to replicate physiological hemodynamic conditions inside the phantoms. A sketch of the circuit is shown in Figure 2.

A versatile piston pump, previously presented in Vignali et al. (2022), along with a mechanical heart valve, was used to generate a pulsatile flow rate. The outlets of the phantoms were connected to pressure-regulated chambers (hybrid units), designed to replicate Windkessel effect. Each hybrid unit, as detailed in Bardi et al. (2023), consists of an acrylic cylindrical chamber where the pressure is pneumatically controlled by solenoid valves connected to a vacuum (−0.5 kPa) and a pressure line (3 kPa). The pressure, measured at the bottom of the chamber, is regulated by a PID controller. The setpoint pressure is dynamically computed at each time step based on the latest flow rate measurement, following a 3-Element Windkessel (3WKM) model. Considering that the aortic model was assumed to be at diastolic pressure, the Windkessel parameters (proximal resistance $\left(R_{p}\right)$ , distal resistance $\left(R_{d}\right)$ , capacitance (C) , and ground pressure $\left(P_{0}\right)$ ) were tuned to achieve a pressure range between 0 and 40 mmHg for each test case.

A CompactRIO controller (cRIO-9047, NI) was used to control the system and to acquire the sensor data. Clamp-on ultrasonic flow meters (Sonoflow CO.55/100 V2.0 - CO.55/190 V2.0, Sonotec) were used to measure the flow rates at the inlet and the outlets of the AAA models. The gains of each ultrasonic flow meter were calibrated by using the encoder of the piston pump, with the same working fluid under pulsatile flow conditions; a delay ranging between 20 ms and 26 ms was found. The pressure inside the hybrid units was monitored through IFM Electronic PA3509 sensors and additional clinical pressure transducers (TruWave, Edwards) were used to monitor the inlet and outlet pressures. All data were logged with a frequency of 5 kHz.

### 2.3 PIV setup

A mixture composed of 60% of glycerol and 40% of water can be used to match the refractive index of Sylgard. However, 22% of urea was added to water - glycerol mixture (62/38) to also maintain the density ρ and the dynamic viscosity μ of the blood (Brindise et al., 2018). The phantoms were immersed in a box filled with the index matched fluid. The flow was seeded with hollow glass spheres of 10 μ m diameter and a density of 1,100 ${\text{kg/m}}^{3}$ . A pulsed high-power LED system (IL-106X LED Illuminator HardSOFT) was used as the illumination source and a fiber optic line light (Schott A08589) equipped with a cylindrical lens was employed to create a light sheet with a thickness ranging from 1 to 2 mm. Images were acquired with a resolution of 1920 × 1200 and 12-bit format. The camera (acA 1920-155 μm - Basler Ace camera) has a pixel size of 5 μm and, and was equipped with a 35 mm lens (RICOH FL-BC3518-9M). The camera exposure time was set to 13000 μs allowing a minimum inter-frame time of 80 μs . The Transistor-Transistor Logic (TTL) signals were generated with an NI9401 module via an FPGA routine embedded in the control software of the HMCL to start the exposure and trigger the illuminator’s double light pulse. The pulse separation time is controlled by the illuminator itself. A breakdown of the cost of the PIV system components is 11500 €, and the list is: Illuminator (4000 €), Line light (450 €), Cylindrical lens (150 €), Camera (900 €), Camera lens (1000 €), FPGA processor (4000 €), and Trigger generation module (1000 €).

The LED pulse-width (τ) and the pulse separation time (Δt) were varied to find the minimum possible illumination time and the maximum duty cycle (τ/Δt) . The duty cycle corresponds to the ratio of the streaking distance during the exposure and the particle displacement. Two pulse widths (10 and 20 μs ) and two pulse separation times (200 and 400 μs ) were evaluated.

The four setting configurations are.

• C1 : τ = 10 μs ; Δt = 200 μs

• C2 : τ = 10 μs ; Δt = 400 μs

• C3 : τ = 20 μs ; Δt = 200 μs

• C4 : τ = 20 μs ; Δt = 400 μs

For each experiment, 50 cardiac cycles were reproduced, and the last 25 cycles were used to acquire the images, namely, 25 images per cardiac cycle (31 ms between each image pair).

Two specific regions of interest (ROIs) were considered for each model, i.e., one proximal to the inlet $\left(ROI_{1}\right)$ and one centered at the bulge of the aneurysm $\left(ROI_{2}\right)$ as reported in Figure 2. In addition, the system was used to capture the pathlines for the same time instants to have a qualitative real-time visualization of the flow field. This was done by using continuous illumination and keeping the camera exposure time at 13000 μs .

### 2.4 Experimental test cases

For the patient-specific AAA model, Doppler-echographic *in-vivo* flow measurements were used to define the flow profile $\left(F_{PS}\right)$ . For the idealized axisymmetric AAA model, the $F_{PS}$ was scaled to define three flow profiles, corresponding to high $\left(F_{H}\right)$ , medium $\left(F_{M}\right)$ , and low $\left(F_{L}\right)$ amplitudes. For each flow condition, the peak Reynolds, the time-averaged Reynolds, and the Womersley (Equation 1) numbers were calculated:

$$Re_{\max}=\frac{U_{\max}d}{\nu }\;\;\langle Re\rangle =\frac{\langle U\rangle d}{\nu }\;\;\alpha =\frac{d}{2}\sqrt{\frac{2\pi }{T\nu }}$$ (1)

where $U_{\max}$ and ⟨U⟩ are the maximum and the average velocity, both derived from the inlet flow rate, d is the inlet diameter (20 mm) , ν is the kinematic viscosity $\left(3.82\;mm^{2}/s\right)$ and T is the cycle period (0.8 s) .

These parameters, along with the 3WKM parameters for the outlet boundary conditions for each flow profile are reported in Table 1.

**TABLE 1:** 3WKM parameters, velocity, Reynolds and Womersley numbers for each flow profile. $R_{p}$ and $R_{d}$ , are expressed in (Kg $m^{-4}$ $s^{-1}$ ), C is expressed in ( $m^{4}$ $s^{2}$ ${kg}^{-1}$ ), $U_{\max}$ and ⟨U⟩ are expressed in (m/s).

AAA model	Flow profile	$R_{p}$	C	$R_{d}$	$U_{\max}$	⟨U⟩	$Re_{\max}$	⟨Re⟩	α
Idealized	$F_{L}$	1.5e7	4e -9	9e8	0.26	0.06	1,367	312	14.3
	$F_{M}$	1.5e7	6e -9	4.5e8	0.44	0.09	2275	468	14.3
	$F_{H}$	1.2e7	8e -9	4e8	0.54	0.11	2815	578	14.3
Patient-Specific	$F_{PS}$	4e7	2e -9	6e 7	0.42	0.1	914	214	9.2

### 2.5 Data processing

**FIGURE 3:**
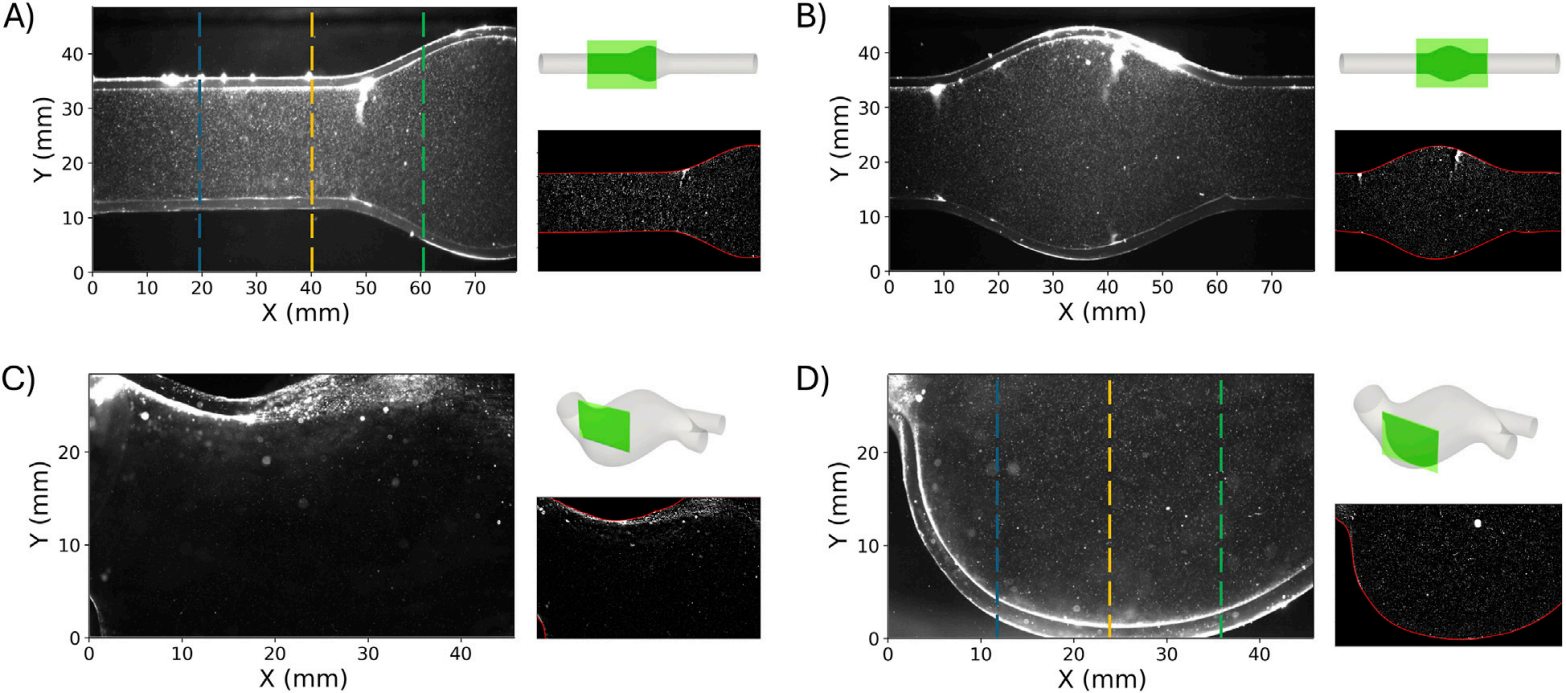
$ROI_{1}$ and $ROI_{2}$ for the idealized **(A, B)** and patient-specific **(C, D)** AAA model, respectively. For each ROI, the original image, with the location scheme and the filtered image are reported. The vertical dashed lines **(A–D)** represent the three different longitudinal locations used to compute the deformation according to Equation 2.

The flow and pressure sensors data were low-pass filtered with a cutoff frequency of 25 kHz. The acquired 25 cycles were considered to obtain a phase average and the corresponding standard deviation. The difference between the flow rate at the inlet and the sum of the outlets was also computed. Concerning the PIV image analysis, a specific pre-processing was applied to enhance the quality of the cross-correlation and to improve the subsequent velocity reconstruction. In particular, the following operations were imposed: background subtraction, intensity rescaling, equalization via contrast limited adaptive histogram processing (CLAHE), Gaussian filtering and edge extraction. In particular, the background was evaluated by starting from the average image and subtracted to the original for each ROI. Then, the intensity of the image was scaled between the 20*th* and 99.5 percentile of the intensity values to extract the inner edges of the phantom. Subsequently, CLAHE and a Gaussian filter were applied to improve the contrast and reduce the noise. At last, the edges of the phantom in each ROI were manually initialized, and then, an accurate segmentation was performed through an active contour algorithm. The results in terms of image pre-processing for each ROI are presented in Figure 3. The obtained contours were finally used to mask the images and to evaluate the deformation of the phantoms at three different longitudinal locations (x) (see Figures 3A, D) by calculating the relative circumferential variation (δ(x,t)) with respect to the dimension at the diastolic time $\left(t_{0}\right)$ , according to:

$$\delta \left(x,t\right)=Y\left(x,t\right)-Y\left(x,t_{0}\right)$$ (2)

Given the limited field of view, Y corresponds to the cross sectional diameter in case of idealized model (Figure 3A), while it corresponds to the vertical dimension in case of patient-specific model (Figure 3D). Since for the idealized model the cross section was circular, Equation 2 was normalised with respect to $Y\left(x,t_{0}\right)$ and reported in percentage.

After image processing, the velocity field was computed using the open-source software OpenPIV (Liberzon et al., 2020; Ben-Gida et al., 2020). The velocity field was calculated using the FFT-based correlation algorithm with a window deformation approach, which involved 3 passes and interrogation areas of 128 × 128, 64 × 64 and 32 × 32 pixels with a 50% overlap. The image resolution for the idealized AAA model and the patient-specific one were 24.6 px/mm and 41.6 px/mm, respectively. The vector validation process consisted of verifying that the global velocity threshold (set at five times the standard deviation) and the local median threshold (set at three times the median) were met. Additionally, the validation ensured that the signal-to-noise ratio was above a value of 1.25. The rate of invalid vectors detected through this process ranged between 0.5% and 3% for the idealized model and between 1% and 9.5% for the patient-specific model.

## 3 Results

### 3.1 Idealized AAA model

**FIGURE 4:**
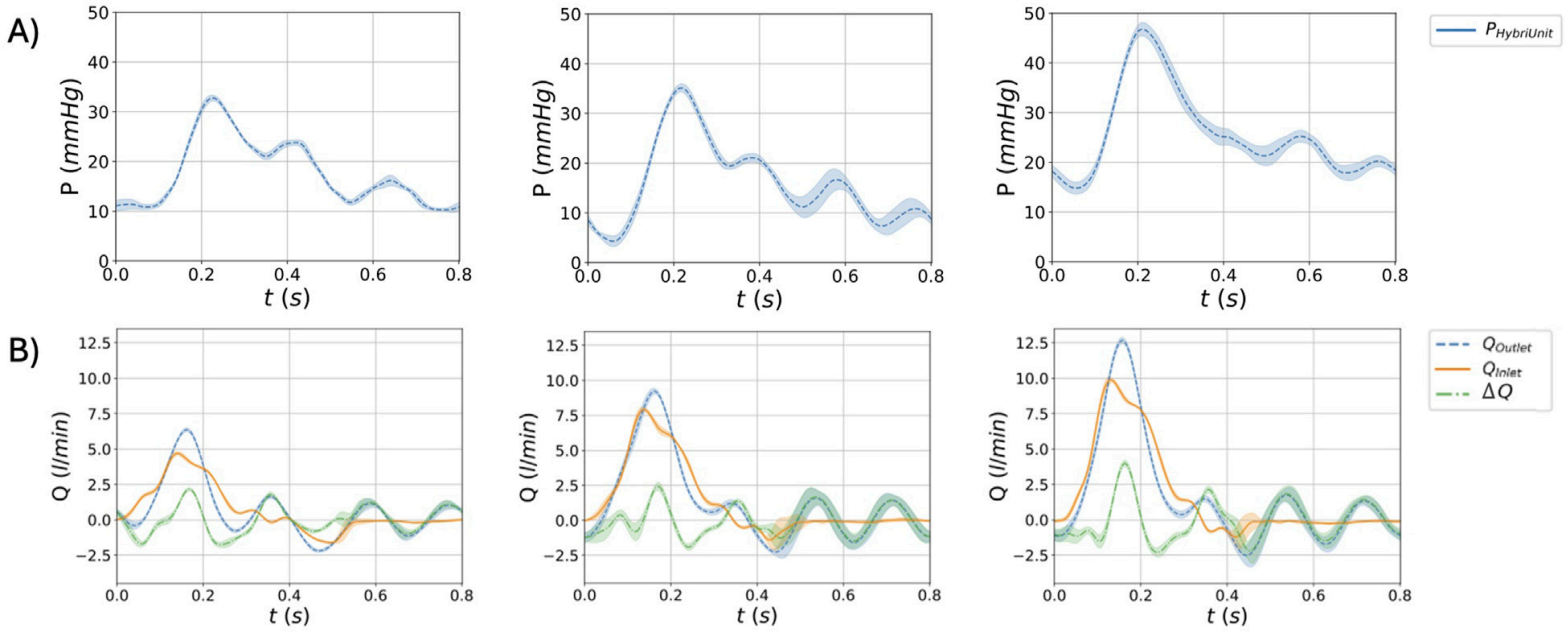
Waveforms for the idealized AAA model for the three flow conditions $F_{L}$ , $F_{M}$ , $F_{H}$ . Pressure in the hybrid unit **(A)**. Flow rate at the inlet, outlet, and their difference Δ Q **(B)**. Results are reported in terms of mean ± sd.

The flow rates measured at the inlet and outlet sections for the three test cases ( $F_{L}$ , $F_{M}$ , $F_{H}$ ) are shown in Figure 4B, including their difference. Figure 4A depicts the outlet pressure imposed by the hybrid unit. All the results are reported on a single cardiac cycle as mean ± sd.

**FIGURE 5:**
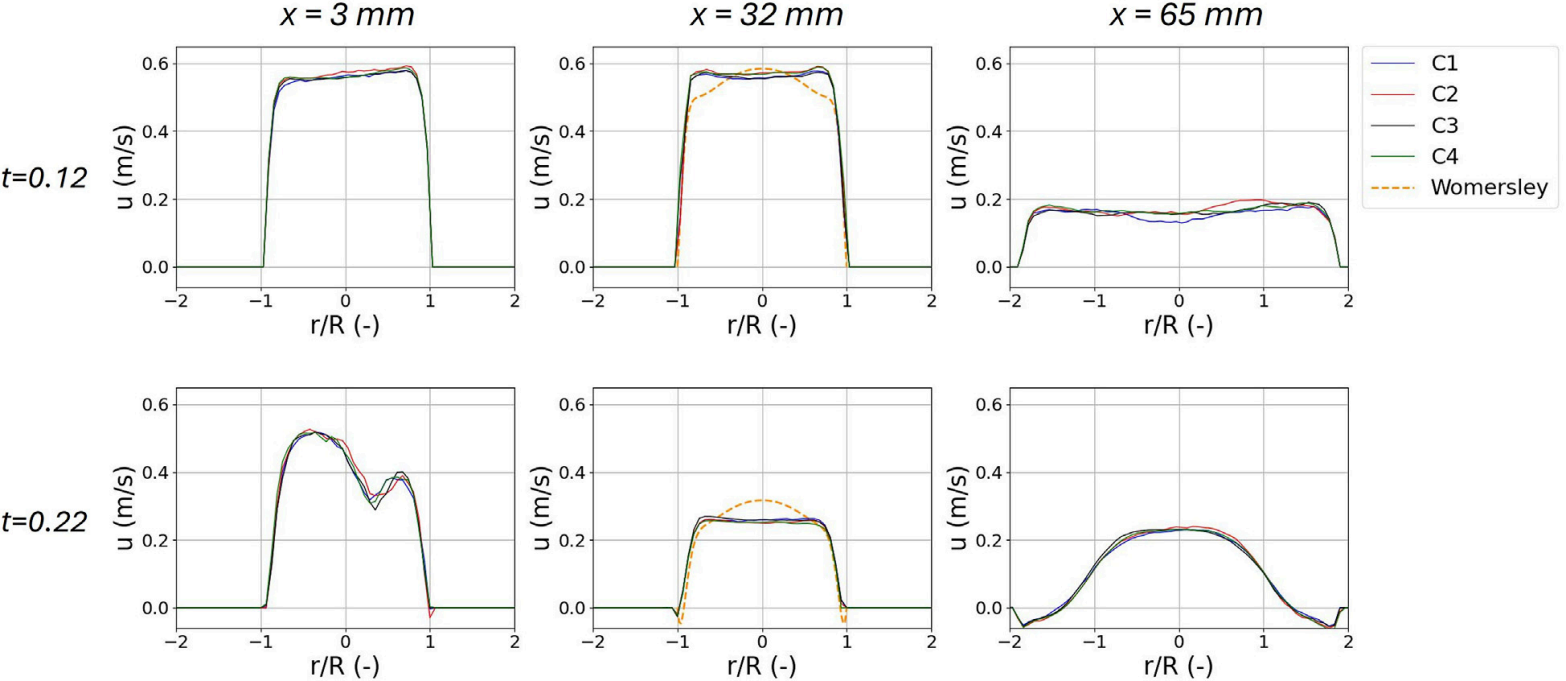
Phase averaged axial velocity profiles (u) in the idealized AAA model at three different x locations (3, 32, and 65 mm) of $ROI_{1}$ at peak systole (0.12 s) **(A)** and late systole (0.22 s) **(B)**. Results are reported for the four illumination settings in the $F_{H}$ flow configuration. For the x=32 mm location, the Womersley velocity profile (dotted line), computed based on the inlet flow rate, is also reported.

Table 2 summarises the δ at the peak systole for the three longitudinal locations reported in Figure 3A. The phase averaged axial component of the velocity u at different longitudinal locations of $ROI_{1}$ (3, 32, 65 mm) are reported in Figure 5 for each of the PIV setting configurations. Results are reported at peak systole and late systole (Figure 5).

**TABLE 2:** Deformation parameter δ at peak systole measured at different longitudinal position and flow conditions.

Flow profile	δ @20 mm	δ @40 mm	δ @61 mm
$F_{L}$	0.6%	1.0%	1.5%
$F_{M}$	0.8%	1.1%	2.0%
$F_{H}$	1.4%	2.0%	3.0%

For the x=32 mm location, the Womersley velocity profile (dotted line), computed based on the inlet flow rate, is also reported. Additionally in Supplementary Figure S1 we extended this comparison to the three flow rate conditions and to the five instants of the cardiac cycle.

**FIGURE 6:**
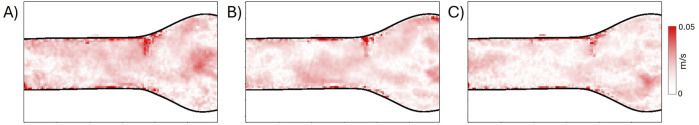
Differences of the velocity field at the systolic peak for the $F_{H}$ flow configuration between $C_{1}-C_{4}$ **(A)**, $C_{2}-C_{4}$ **(B)**, and $C_{3}-C_{4}$ **(C)** PIV setting configurations.

Figure 6 shows the differences between the PIV setting configurations with respect to $C_{4}$ . Given the maximum difference achieved in these maps of 0.05 m/s, the subsequent results are presented only for the $C_{4}$ setting for brevity.

**FIGURE 7:**
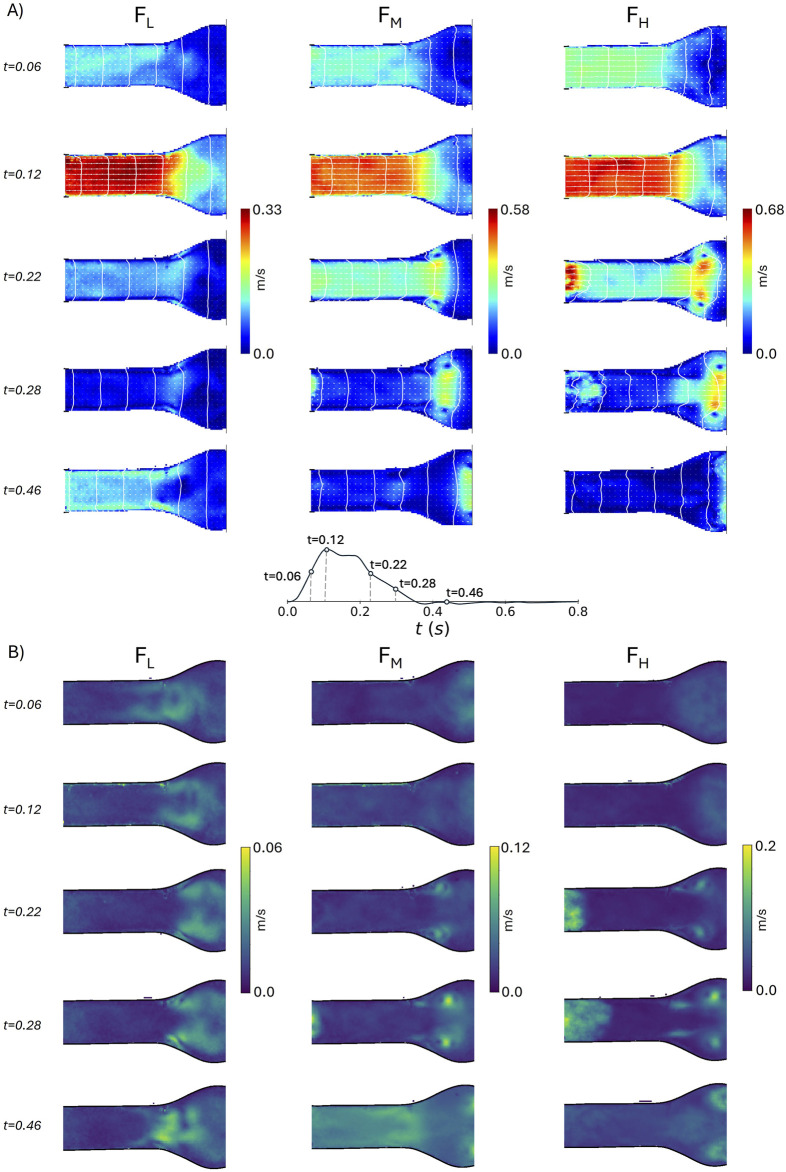
Velocity magnitude field with velocity vectors **(A)** and root mean square of velocity fluctuations **(B)** in the idealized model at $ROI_{1}$ for the three different flow conditions $F_{L}$ , $F_{M}$ , $F_{H}$ at five instants of the cardiac cycle (0.06, 0.12, 0.22, 0.28, and 0.46 s).

**FIGURE 8:**
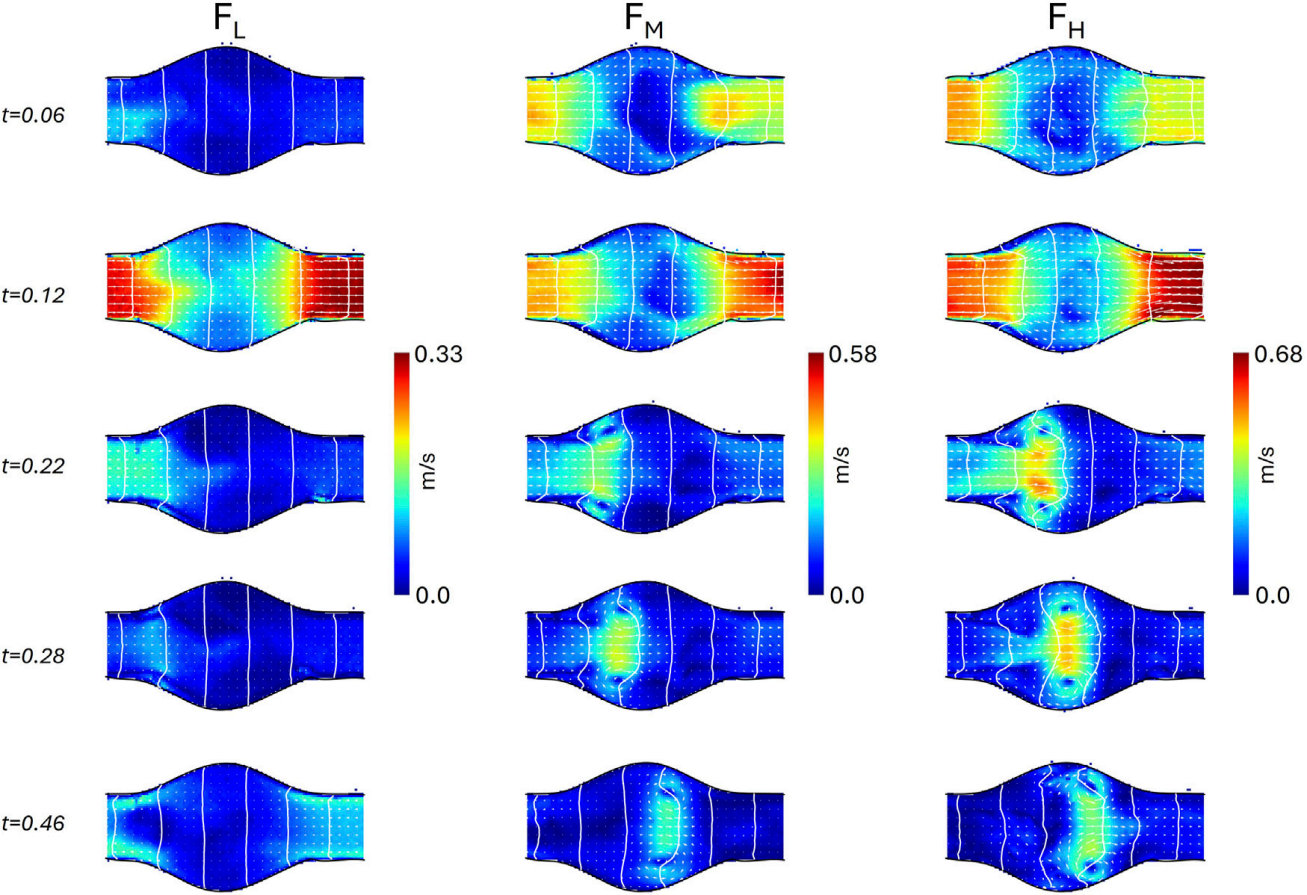
Velocity magnitude field with velocity vectors in the idealized model at $ROI_{2}$ for the three different flow conditions $F_{L}$ , $F_{M}$ , $F_{H}$ at five instants of the cardiac cycle (0.06, 0.12, 0.22, 0.28, and 0.46 s).

Figures 7A, 8 present the velocity vectors and the velocity magnitude fields for each flow condition. Five time-frames (at 0.06, 0.12, 0.22, 0.28, and 0.46 s), obtained under the $C_{4}$ illumination condition are displayed, for $ROI_{1}$ and $ROI_{2}$ respectively. Figure 7B also shows the root mean square of the velocity fluctuations, calculated as $u_{\mathrm{rms}}^{\prime }=\sqrt{\bar{u_{x}^{\prime 2}}+\bar{u_{y}^{\prime 2}}}$ , where $\bar{u_{x}^{\prime 2}}$ and $\bar{u_{y}^{\prime 2}}$ are the squared standard deviations of the two velocity components.

**FIGURE 9:**
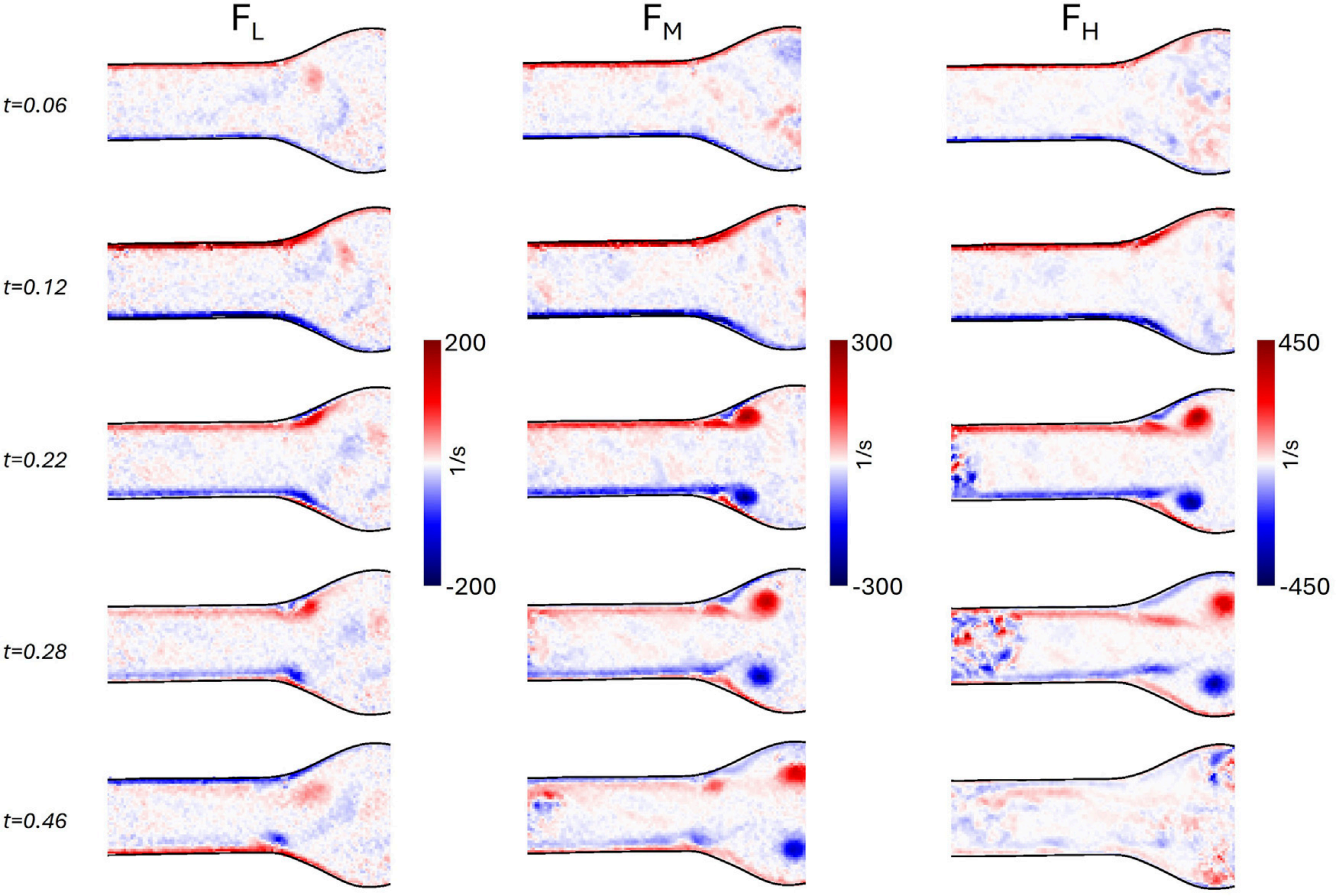
Vorticity field in the idealized model at $ROI_{1}$ for the three different flow conditions $F_{L}$ , $F_{M}$ , $F_{H}$ at five instants of the cardiac cycle (0.06, 0.12, 0.22, 0.28, and 0.46 s).

**FIGURE 10:**
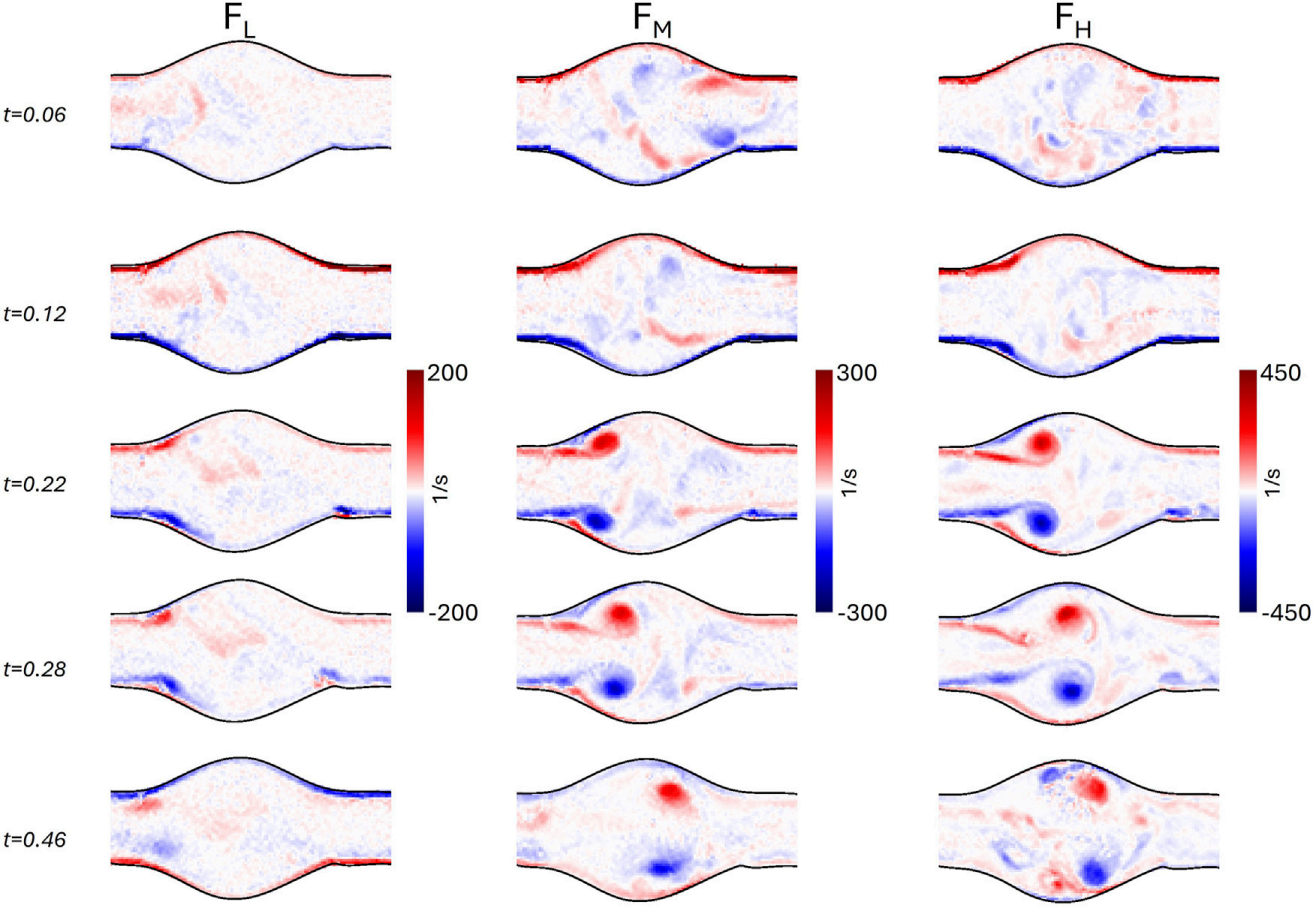
Vorticity field in the idealized model at $ROI_{2}$ for the three different flow conditions $F_{L}$ , $F_{M}$ , $F_{H}$ at five instants of the cardiac cycle (0.06, 0.12, 0.22, 0.28, and 0.46 s).

Analogously, the vorticity magnitude field is shown in Figures 9, 10, for $ROI_{1}$ and $ROI_{2}$ respectively.

**FIGURE 11:**
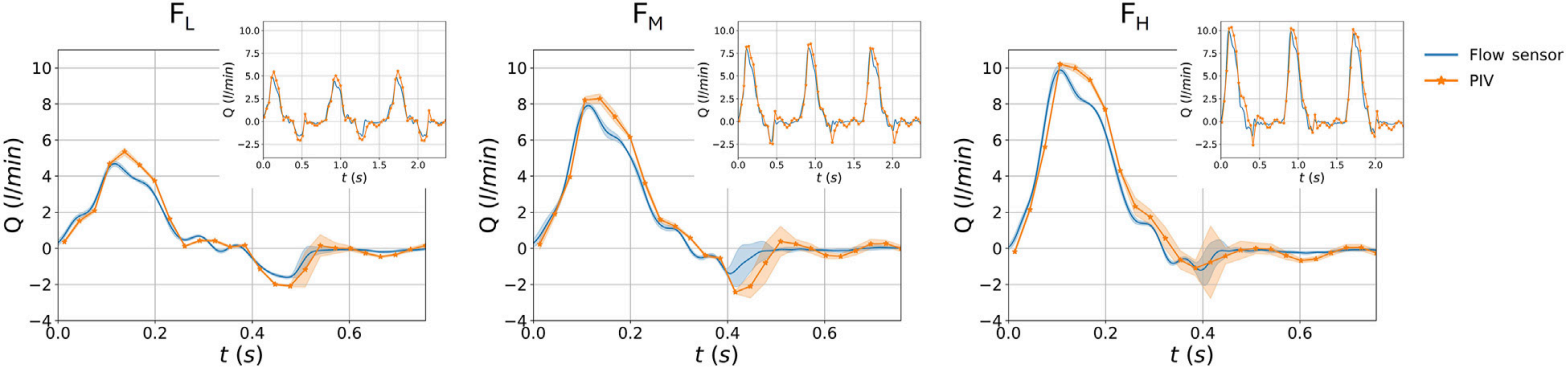
Comparison between the sensor measured flow rate, and the PIV derived flow rate, for the three flow rate conditions $F_{L},F_{M}$ and $F_{H}$ . Results are reported in terms of mean ± sd and three cardiac cycles in the corresponding sub panels.

The longitudinal component of the velocity at the inlet of $ROI_{1}$ (between 8 and 12 mm) was averaged along the *x*-axis. The resulting velocity profile was then integrated to obtain the flow rate Q(t) , assuming axial symmetry of the flow. This can be expressed as:

$$Q\left(t\right)=\pi \int_{-R}^{R}u_{x}\left(r,t\right)\cdot rdr$$

where $u_{x}\left(r,t\right)$ is the velocity profile as a function of the radial position r and time t . The computed flow rate was compared with the measurements from the inlet flow rate sensor for all test cases. The flow rate sensor measurements and the flow rate derived from PIV are compared in Figure 11; the first three periods and the phase averages (with the standard deviations) are shown.

### 3.2 Patient-specific AAA model

**FIGURE 12:**
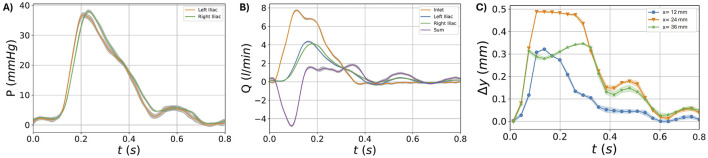
Waveforms for the patient-specific model. Pressure in the two hybrid units **(A)**. Flow rate at the inlet, outlets (left and right iliac), and and their difference Δ Q **(B)**. Deformation of the phantom at the three different locations of $ROI_{2}$ (12, 24, 36 mm) **(C)**. Results are reported in terms of mean ± sd.

**FIGURE 13:**
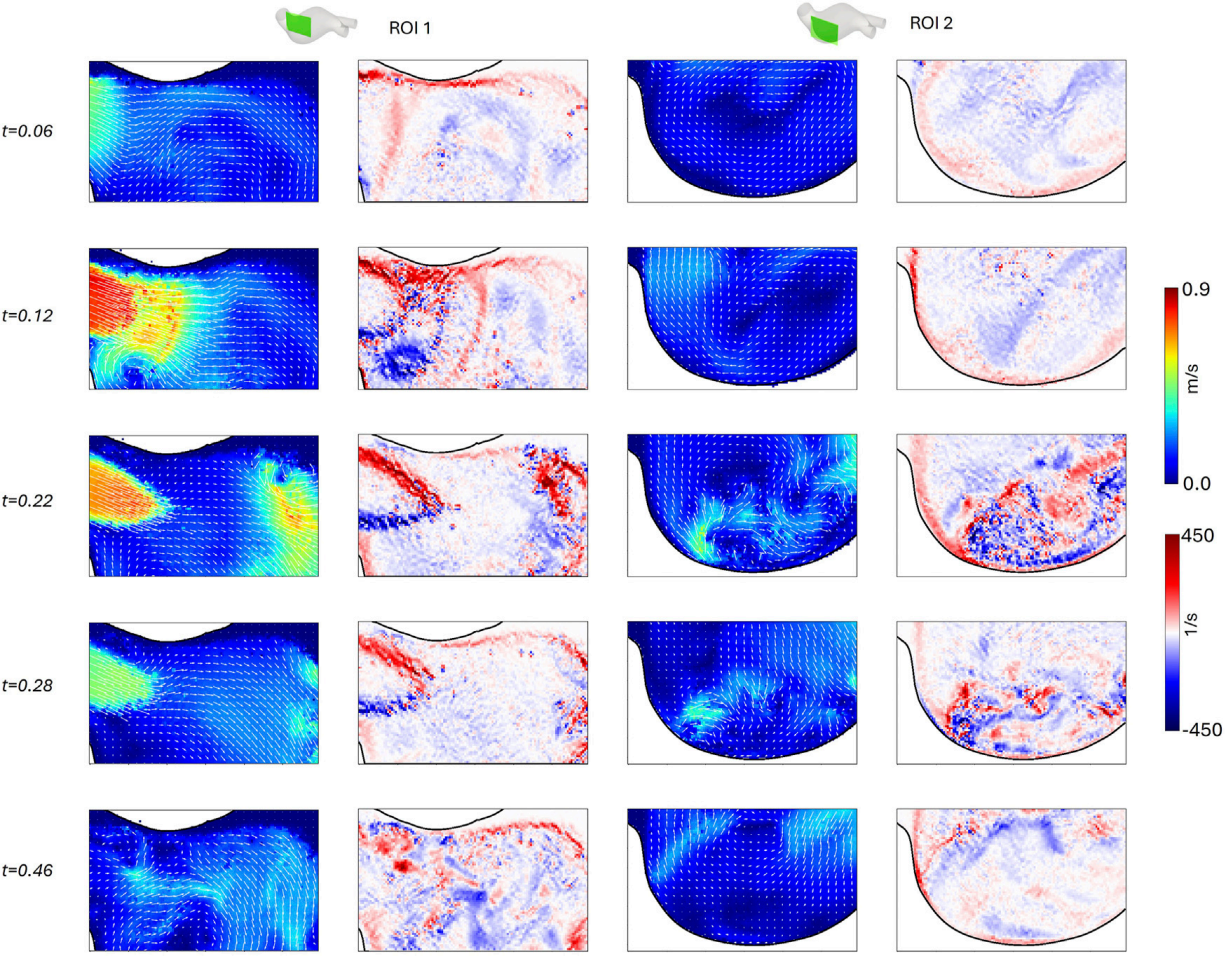
Velocity magnitude field with velocity vectors and vorticity field for $ROI_{1}$ and $ROI_{2}$ of the patient-specific model, at five instants of the cardiac cycle (0.06, 0.12, 0.22, 0.28, and 0.46 s).

Figure 12 depicts the phase-averaged waveforms of the flow rates at inlet, outlets and their balance; the pressure in the hybrid units, and deformations at three different longitudinal locations on $ROI_{2}$ for the patient-specific AAA model. The obtained velocity and vorticity fields (under the $C_{4}$ illumination condition) at five instants of the cardiac cycle (0.06, 0.12, 0.22, 0.28, and 0.46 s ) are shown in Figure 13, for $ROI_{1}$ and $ROI_{2}$ respectively.

## 4 Discussion and conclusion

MCL systems have gained increasing popularity in the experimental analysis of cardiovascular systems due to their ability to replicate hemodynamic conditions with high fidelity (Zimmermann et al., 2021; Vignali et al., 2022; Agrafiotis et al., 2024). Integrating a high-fidelity HMCL with a PIV system serves two crucial purposes. Firstly, it provides a comprehensive benchmark for validating *in silico* data from numerical approaches (Mariotti et al., 2020; Lan et al., 2022; Fanni et al., 2023). Secondly, this setup can be used to gain new insights by replicating *in-vitro* multiple hemodynamic conditions as observed in clinical environments and is a valuable tool for enhancing the accuracy of other velocity reconstruction methods, such as Color-Doppler imaging (Antonuccio et al., 2022). Additional value is added when a compliant phantom is used to mimic patient-specific cardiovascular conditions (Fanni et al., 2020; Wu et al., 2022; Antonuccio et al., 2023; Moravia et al., 2023) for a deeper understanding of hemodynamic parameters such as pressure, flow, velocity fields, and vessel deformation.

The integrated PIV-HMCL system presented in this work is particularly effective due to its ability to operate under controlled and highly reproducible pressure conditions, enabled by using hybrid chambers (Bardi et al., 2023). Moreover, the feasibility of using a high-power LED-PIV system promotes the spread of *in-vitro* studies in cardiovascular research (Cierpka et al., 2021; Torta et al., 2024). Our low-cost setup significantly reduces the investment required by conventional PIV systems up to approximately one hundred thousand euros by eliminating the need for dedicated high-speed cameras, high-energy laser sources, and specialized PIV synchronization units. The choice of an LED light source offers the crucial advantage of being safer and less hazardous than lasers used in traditional PIV systems, which require specific precautions and procedures as envisaged by the international standard IEC 60825–1.

This study is the first to explore the feasibility of low-cost LED-PIV measurements for the *in-vitro* characterization of abdominal aorta flows under fully controlled patient-specific pulsatile flow and pressure conditions. Specifically, testing was conducted on both an idealized AAA model and a patient-specific one.

Concerning the idealized model, Figure 4 represents the results in terms of flow, pressure, and deformations. In particular, the results demonstrate that the HMCL could replicate three different fluid dynamic conditions, both in terms of pressure and flow (Figures 4A, B) waveforms. The obtained pressure values were consistent with the physiological range of pressures in the abdominal aorta (Amanuma et al., 1992). Moreover, the results demonstrate the system’s repeatability, given the standard deviation range observed in the plots. The phantom’s deformability is responsible for differences between inlet and outlet flow waveforms. These effects were quantified by the δ parameter at the systolic flow peak (Table 2). These effects are more evident in the third location, which is the most distant from the hydraulic connector acting as a constraint. The reported results indicate that the aortic phantoms faithfully replicated the realistic physiological behavior of an *in-vivo* vessel (Bracco et al., 2023; Derwich et al., 2023). Moreover, based on the pressure measurements and deformations observed in the idealized phantom, it was possible to determine the distensibility (pressure-strain elastic modulus) at various longitudinal locations. Specifically, we found distensibility values of $3.8\times 10^{5}\;{\text{Nm}}^{-2}$ and $2.8\times 10^{5}\;{\text{Nm}}^{-2}$ at x=40 mm and x=61 mm respectively. These values are consistent with distensibility range reported by Wilson et al. (2003) $\left(2-4.3\times 10^{5}\;{\text{Nm}}^{-2}\right)$ .

Concerning the effect of the illumination settings on the velocity obtained from the PIV data processing, the four configurations tested did not reveal relevant differences. This is evident from the three axial velocity profiles of Figure 5 for the $F_{H}$ flow condition at the peak systole (0.12 s) and late systole (0.22 s). Due to the minimal differences in the velocity profiles, the phase-averaged velocity magnitude at peak systole was compared to that of the $C_{4}$ configuration in $ROI_{1}$ (Figure 6) allowing a spatial visualization of the regions affected by the highest differences. These results revealed a maximum difference of 0.05 m/s, confined to the phantom wall; this local effect is likely linked to light scattering (Figure 3A). Compared to the reference settings $C_{4}$ ( τ=20 μs and Δt=400 μs ), neither reducing the pulse width to 10μs nor doubling the duty cycle showed any significant difference. This suggests that it would be possible to measure even higher velocities using a pulse width of 10 μs and pulse separation 100 μs (duty cycle of 10%). Additionally, by inspecting Supplementary Figure S1, we can notice that the profiles corresponding to the $C_{4}$ configuration reveal differences with the Womersley profile by increasing the flow amplitude. The behaviour depends on the fact that $F_{L}$ , characterized by a lower Reynolds, is less affected by the connection between the phantom and the rigid adapter. In the cases of $F_{M}$ and $F_{H}$ , its presence prevent the flow from fully developing. The behaviour appears to be influenced also by the cardiac phase. In fact, while at the systolic peak (t = 0.12 s) a good agreement was observed, at late systole and the early diastole, an asymmetry in the flow profile is visible. This is particularity true for the $F_{H}$ profile. These behaviours are in line with those reported in Figure 7 where the root mean square of the velocity fluctuations are higher.

Although the geometry of the idealized AAA model is the same as the geometry presented in Salsac et al. (2006b), and similar Reynolds and Womersley numbers were used, a point-wise quantitative comparison of velocity and vorticity fields is not feasible. Indeed, we imposed different systole-to-diastole time ratios, the backflow was only due to the regurgitation of the heart valve, and the phantom was flexible. Nonetheless, it is possible to qualitatively compare the velocity (Figures 7, 8) and the vorticity (Figures 9, 10) fields. In particular, the flow field of $F_{H}$ is consistent with the observations of Salsac et al. (2006b). Specifically, during the systolic acceleration phase (0.12 s), the flow remains parallel to the wall boundary, and it reverses close to the wall in the proximal half of the aneurysm bulge at the beginning of the deceleration phase (0.22 s). This creates a vortex ring that travels downstream, impinging the distal neck, and creating secondary vortices in the bulge. The same flow patterns were also observed in Bauer et al. (2020), where magnetic resonance velocimetry acquisitions were compared with numerical simulations.

According to Yip and Yu (2001), this flow regime, also observed in $F_{M}$ , is characterized by a cyclic transition to turbulence within the bulge during the deceleration phase. Conversely, in $F_{L}$ , the vortex remains stationary, and the flow stays laminar. This behavior is confirmed by the heatmaps of the root mean square of the velocity fluctuations, as depicted in Figure 7B. The velocity fluctuations reflect contributions from both turbulence and cycle-to-cycle variations; however, during systole, the influence of cycle-to-cycle variations is minimal, as indicated by the standard deviation in flow rate measurements (Figure 4B). At t=0.06 s, the highest $u_{\mathrm{rms}}^{\prime }$ values are concentrated in the bulk of the aneurysm and turbulence intensity reaches is minimum at peak systole. During the deceleration phase high values of $u_{\mathrm{rms}}^{\prime }$ are localized at the inlet, where the connection between the phantom and the rigid adapter disrupts the flow, and at the core of the vortices. In contrast, at t=0.46 s, when the standard deviation in flow rate reaches its maximum (Figure 4B), $u_{\mathrm{rms}}^{\prime }$ is not localized but generally elevated across the flow field.

Finally, the flow rate calculated from the axial velocity and the flow rate measured with the sensor (Figure 11) were found consistent for all three flow rate conditions. The maximum error was found to be less than 2 L/min. For all flow conditions, there is a visible difference at peak systole. This difference is likely due to the velocity being taken 3 cm from the rigid adapter, where the inlet flexible part acts as a compliance. Other discrepancies could be due to the inaccurate hypothesis of perfectly axial symmetrical flow and the accuracy of the flow sensors in measuring flow rates, especially for lower flow rates.

Considering then the patient-specific model, the waveforms of Figure 12 **(A)** show a physiological behavior in terms of pressures. Moreover, by observing the mean and standard deviation values of pressure, we can confirm the reproducibility of the pressure conditions prescribed by the HMCL. As already observed in the AAA model, the Δ Q quantities reflect the model deformation as a consequence of its compliance (Figure 12B). The same behavior is reflected by the δ value variation during the cardiac cycle (Figure 12C). Since the patient-specific geometry has an intrinsic complexity with a twisted centerline and a high dilation ratio (i.e., the ratio between the inlet diameter and the maximum diameter) as a consequence of the aneurysm presence, a more complex flow field was captured (Figure 13). Indeed, the abrupt change in diameter at the inlet section induces a jet-like flow at the systolic phase (t = 0.12 s), as highlighted in $ROI_{1}$ . During the deceleration phase (t = 0.22 s), a vortex forms in the bulge of the aneurysm. This vortex likely travels perpendicularly to the acquiring plane of $ROI_{2}$ , characterized by low in-plane velocity values across the whole cardiac cycle. Previous studies (Deplano et al., 2016; Chen et al., 2014) have highlighted the importance of measuring the out-of-plane velocity component for properly describing the hemodynamics in complex patient-specific AAA geometries. In our experiments, the out-of-plane velocity component is likely significant due to a misalignment between the proximal neck and the iliac bifurcation, and for the presence of the AAA bulge. Stereoscopic PIV should be considered as a future development of our setup to understand the complete flow behavior in the bulge.

Additionally, the thickness of the light sheet and light reflections constrained the choice of acquiring planes. In the case of complex geometry such as the one of this study, the definition of a suitable PIV plane is not a trivial task due to the light incidence on the curved surface (Ronneberger et al., 1998; Edwards et al., 2020). As shown in Figures 3C,D, a noticeable reflection was observed in the patient-specific model despite the CLAHE filtering. This blurring effect likely compromised the accuracy of the velocity field reconstruction, making the results in the small region near the curved wall unreliable. The issue of spurious reflections could potentially be mitigated by using fluorescent particles combined with an appropriate filter.

This work showed that high-power LED-illuminated PIV is a viable and affordable alternative to a standard laser PIV system to study large blood vessels’ hemodynamic *in-vitro*. LED-PIV is safe to use in both educational/training and clinical settings. In the CVD research context, as numerical models keep gaining importance, the need for accurate and reliable results has become increasingly more relevant (Viceconti et al., 2021; Lan et al., 2022). The present study addresses this need by providing experimental data that can be used to evaluate and refine numerical models.

## Data Availability

The raw data supporting the conclusions of this article will be made available by the corresponding author upon reasonable request.
