# Supplementary material for: A hybrid mock circulatory loop integrated with a LED-PIV system for the investigation of AAA compliant phantoms
Source: Front Bioeng Biotechnol. 2024 Oct 10;12:1452278. doi: 10.3389/fbioe.2024.1452278 (PMC11499900; doi:10.3389/fbioe.2024.1452278)
Supplement: Supplementary file 1 [file DataSheet1.pdf]

Supplementary Material

1 SUPPLEMENTARY TABLES AND FIGURES

In Figure S1 the measured axial velocity profile (u) in the idealized AAA model at $x = 32$ mm of ROI_1 is compared with the Womersley velocity profile (dotted line), which was computed based on the inlet flow rate. The three different flow conditions F_L, F_M, F_H at five instants of the cardiac cycle (0.06, 0.12, 0.22, 0.28, and 0.46 s) are shown.

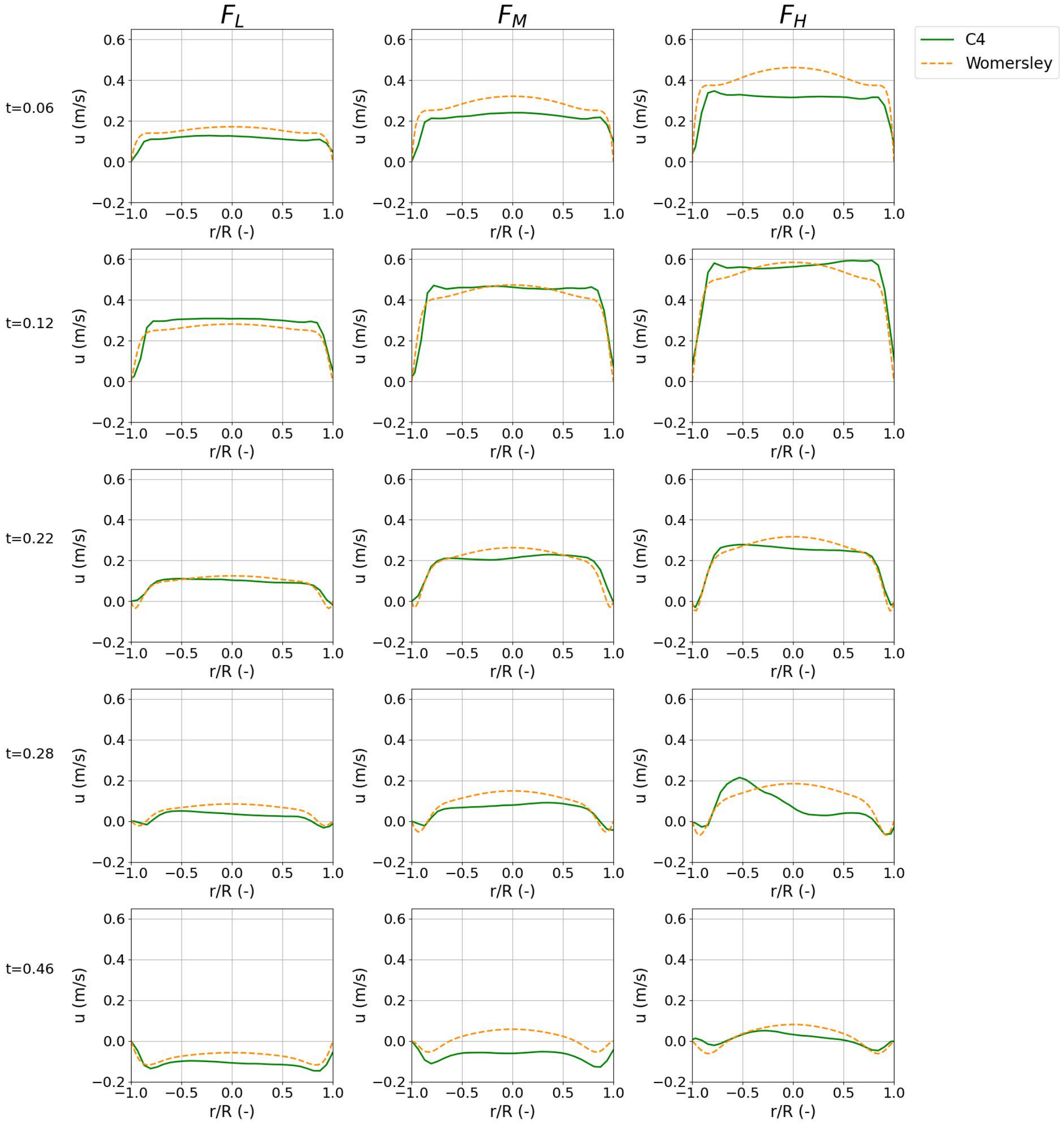

Figure S1. Comparison between the measured axial velocity profile (u) and the Womersley velocity profile (dotted line), computed based on the inlet flow rate, in the idealized AAA model at $x = 32$ mm of ROI_1. The three different flow conditions F_L, F_M, F_H at five instants of the cardiac cycle (0.06, 0.12, 0.22, 0.28, and 0.46 s) are shown.
